# Nano Hotplate Fabrication for Metal Oxide-Based Gas Sensors by Combining Electron Beam and Focused Ion Beam Lithography

**DOI:** 10.3390/mi14112060

**Published:** 2023-11-04

**Authors:** Zhifu Feng, Damiano Giubertoni, Alessandro Cian, Matteo Valt, Mario Barozzi, Andrea Gaiardo, Vincenzo Guidi

**Affiliations:** 1 Istituto Italiano di Tecnologia, Via Morego, 30, 16163 Genova, Italy; 2 Micro-Nano Characterization and Fabrication Facility Unit, Sensors and Devices Center, Bruno Kessler Foundation, Via Sommarive 18, 38123 Trento, Italyacian@fbk.eu (A.C.); mvalt@fbk.eu (M.V.); barozzi@fbk.eu (M.B.); gaiardo@fbk.eu (A.G.); 3 Department of Physics and Earth Science, University of Ferrara, Via Saragat 1, 44122 Ferrara, Italy

**Keywords:** gas sensors, nano heaters, electron beam lithography, focused ion beam, ion beam lithography, power consumption

## Abstract

Metal oxide semiconductor (MOS) gas sensors are widely used for gas detection. Typically, the hotplate element is the key component in MOS gas sensors which provide a proper and tunable operation temperature. However, the low power efficiency of the standard hotplates greatly limits the portable application of MOS gas sensors. The miniaturization of the hotplate geometry is one of the most effective methods used to reduce its power consumption. In this work, a new method is presented, combining electron beam lithography (EBL) and focused ion beam (FIB) technologies to obtain low power consumption. EBL is used to define the low-resolution section of the electrode, and FIB technology is utilized to pattern the high-resolution part. Different Au^++^ ion fluences in FIBs are tested in different milling strategies. The resulting devices are characterized by scanning electron microscopy (SEM), atomic force microscopy (AFM), and secondary ion mass spectrometry (SIMS). Furthermore, the electrical resistance of the hotplate is measured at different voltages, and the operational temperature is calculated based on the Pt temperature coefficient of resistance value. In addition, the thermal heater and electrical stability is studied at different temperatures for 110 h. Finally, the implementation of the fabricated hotplate in ZnO gas sensors is investigated using ethanol at 250 °C.

## 1. Introduction

With the increasing population and industrial production, the monitoring of harmful gases plays a major role for human and environmental health [1]. Several technologies have been investigated to detect gases qualitatively and quantitatively. Among others, chemoresistive gas sensors are widely used due to their great versatility and the potential to exploit a wide range of sensing materials with different surface reactivity and electrical properties [2,3,4]. Metal oxide semiconductors (MOS) are the most long-standing and still the most widely exploited materials for chemoresistive gas sensors due to their low cost, easy fabrication, and stability [5]. Although MOS gas sensors functioning at room temperature are quite desirable because of their low power consumption and comparable gas sensing performances, such as high sensitivity and selectivity [6], they still pose the deadly risk of being affected by fluctuant ambient temperatures, since the semiconductor materials are very sensitive to temperature changes. Generally, the commercial MOS gas sensor devices operate at high working temperatures provided by hotplates. When targeting the application of this platform for portable devices, it is necessary to decrease the power consumption for the heating elements [4,7,8,9]; thus, the power consumption of the hotplate is one of the most significant factors to consider and optimize. Micro-hotplates (MHPs) are widely used to provide high operating temperatures for MOS gas sensor devices. MHPs emit heat through setting up a current to a resistor circuit driven by low levels of power with a very short response time [10]. Developing lower power consumption hotplates for MOS gas sensor devices can expand portable applications and operations at remote off-grid locations [11]. Therefore, maintaining the high operation temperature of the hotplate while decreasing the power consumption is crucially necessary for MOS sensor applications. 

Nowadays, silicon micro hotplates are widely developed due to the vigorous progress of MEMS technology, which allows for particular geometry miniaturization and structure bulk micromachining [12]. The development of suspended membranes or closed membranes can achieve mW-level power consumption for micro hotplates. These MEMS fabrication strategies show magnificent advantages to decrease the size of the hotplates to a micro/nano level based on Si micromachining [9]. For example, H.R. Shwetha et al. achieved a power consumption of 5.85 mW at a temperature of 300 °C with a suspended membrane etched from the backside of the silicon substrate [13]. Furthermore, Bagolini et al. reported that a 3 × 3 mm^2^ microheater fabricated by photolithography could achieve 450 °C with a rising time of 1.06 s and a falling time of 1.14 s from room temperature run by a 107 mW power [14]. Avneet Singh et al. developed a microheater with a 250 × 250 μm^2^ heating area driven by low power consumption (<35 mW) through the bulk micromachining technique [15]. Jianwen Sun et al. investigated a circular structure and an integrated micro-heater when the input voltage was 2 V and the working temperature was 100 °C with a power consumption of 60 mW on a silicon platform [16]. Timo Schössler presented a platinum-resistor micro-hotplate with thermal long-term reliability, when the temperature was around 250 °C and the power was 50 mW [17]. 

However, suspended or closed membranes are easy to break up and their use can be limited. A. Gaiardo and coworkers tested the failure force of a 900 nm thick closed membrane with different planar sizes; the strongest affordable force was 205 mN for a 0.9 × 0.9 mm^2^ membrane layout, and the least resistant layout showed a 37 mN failure force for a 1.74 × 1.74 mm^2^ membrane structure [18]. These membranes are typically so fragile that no further process can be borne on top of it, such as screen printing or sputtering materials. From this, it follows that in many cases, solid substrates have to be used. For example, Y. Wang et al., for their sensors, needed to sputter the heterostructure-sensing material Au/SnO_2_:NiO onto the self-assembled Au nanoparticle arrays. They used the Langmuir–Schaefer technique to transfer the Au nanoparticles to the solid substrate [19]. Phung Thi Hong Van et al. fabricated an effective networked nanowire sensor on a Si/SiO_2_ substrate via on-chip growth. The heater and electrodes were patterned by conventional lithography and lift-off techniques, and the sensing materials were grown by thermal evaporation at 1000 °C on the solid Si-base substrate [20]. Daihua Zhang et al. deposited In_2_O_3_ nanowires onto solid Si wafers covered with 500 nm SiO_2_ and patterned the finger-shaped metal electrodes by photolithography and electron beam evaporation on top of the sensing material [21]. For these reasons, the substrate cannot be a suspended structure membrane; therefore, the only approach to decrease the power consumption is the miniaturization of the heating component on a highly mechanically stable substrate. This approach has been proven to be a valid way to improve power efficiency; due to the small device area that is to be heated up, temperature distribution is well-controlled, and thermal response is fast enough for the devices [22]. 

To increase the power efficiency by miniaturization, non-standard MEMS fabrication methods are required to downsize the hotplate geometry. As is well known, UV photolithography is an ideal MEMS technique for microfabrication in mass production, but it requires mask design processing, and most of the time multiple masks are required, resulting in higher costs and a longer fabrication time of the devices. For initial research and trial activities, it is convenient to adopt maskless fabrication techniques. Electron beam lithography (EBL) is a flexible, maskless and direct writing technique for modern electronics, which offers the versatility to quickly fabricate customized patterns [23]. This technique requires a specific optimization of the processing parameters since they are highly dependent on the design and the substrate. These devices exhibit patterns of varying sizes, such as electrodes and connections with lengths of hundreds of microns and heating elements with dimensions close to 1 μm and spacings of hundreds of nanometers. Standard UV lithography (with mask aligner systems) cannot accurately produce this type of layout, and even EBL has limitations when defining patterns with varying sizes and densities. This limitation comes from the fact that a single recipe cannot be used for both small and large features in a reasonable time of patterning. In particular, beam parameters optimized for small structures would lead to long exposure, while the ones optimized for faster exposures would lead to features much different from those of the designed layout [24]. Supported by these observations, alternative fabrication strategies can be taken into consideration to obtain heaters with sharp edges and nanometric spacings.

Focused ion beam (FIB) instrumentation has been extensively developed and has become an important technique in micro/nano fabrication. FIB systems are widely used not only for characterization with their ability to produce cross sections but also as a fabrication tool due to their capability of high-resolution direct patterning [25,26,27]. Patterning with an FIB by ion sputtering occurs when the material is removed caused by the interaction of high-energy ion beams with the target material. The control of the beam positioning in FIB systems allows direct patterning of the substrate with limited lateral scattering [28]. The high ion doses and limited beam currents required for fine milling make this technique as slow as the high-resolution patterning with EBL. However, compared to EBL, high-resolution FIB patterning has several advantages. Firstly, it does not require coating and development steps, and secondly it is not affected by proximity effects, so sharp corners and higher fill factors can be achieved.

**Figure 1:**
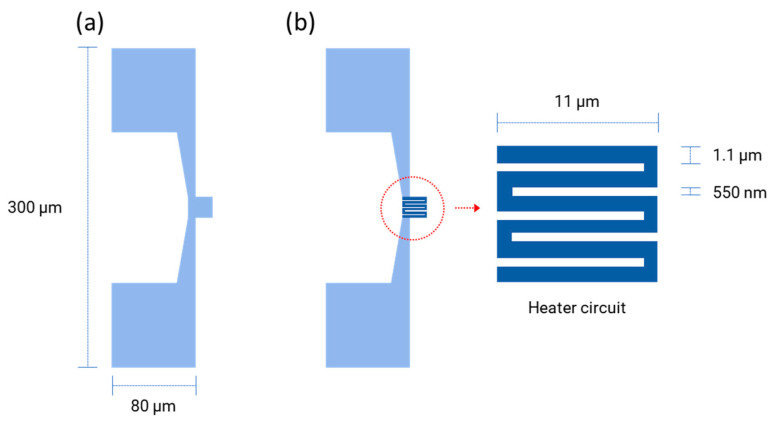
Layout design of the nano-heater structure. (**a**) Layout of electrodes and contacts for EBL patterning; (**b**) Layout of the heating element for FIB patterning.

Combining EBL and FIB techniques is a creative way to improve the efficiency and precision of the nano- and microfabrication processes. EBL can be a fast maskless technique to fabricate large-area and low-resolution patterns, while an FIB can be implemented to shape them into high-resolution structures with dimensions comparable to the nominal ones. In this work, a new approach by combining EBL and FIB techniques is investigated to fabricate the hotplate device for MOS gas sensors (as shown in Figure 1a,b), which is composed of two parts: the loosely distributed micro-level pad parts and the heating circuit part with a dense structure. Since the minimum spacing of the heating circuit elements is 550 nm and the total thickness of the metallic layer is 110 nm, this hotplate is considered to be a nano hotplate (NHP). Here, EBL is used to pattern the micro-pads, and then an FIB is used to define the nano-heating circuit parts, as shown in Figure 1a,b, respectively.

The parameters for EBL are defined by iterative optimization, while the ion beam parameters of an FIB are defined by optimization of the process supported by simulations performed with Stopping and Range of Ions in Matter software (SRIM, ver. SRIM-2013), based on the binary collision approximation Monte Carlo method to study the effects of impinging ions [29,30]. A scanning electron microscope (SEM) is then used to characterize the morphologies, an atomic force microscope (AFM) is used to investigate the surface and the milling depths, and secondary ion mass spectrometry (SIMS) is carried out to determine the cross-sectional ion implantation distribution. Finally, electronic measurements are operated to study the variation in resistance and power consumption at different input voltages.

## 2. Materials and Methods

### 2.1. NHP Substrate Preparation

The silicon-type substrate providing the mechanical support for the NHP is coated with a SiO_2_/Si_3_N_4_/SiO_2_ (ONO) stack structure, which has been shown to be an effective support for zero-stress electrical devices. This substrate preparation is carried out at Sensors and Devices Centre of the Bruno Kessler Foundation (FBK-SD, Trento, Italy). The fabrication is performed according to the process developed at the Micro-nano Characterization and Fabrication Facility (MNF) by Plasma Enhanced Chemical Vapor Deposition (PECVD) and thermal growth [14,18]. The SiO_2_ and Si_3_N_4_ multilayers act as passivation layers for the heating circuit due to their high resistance to electrical conductivity and thermal conductivity.

### 2.2. Heater and Electrode Material Selection

There are a variety of materials used in the fabrication of plate resistor circuits, such as Silicon carbide (SiC), platinum (Pt), poly-Silicon, and Titanium nitride (TiN) [31,32,33]. The key factor for hotplates is the control and measurement of the operating temperature at different input voltages. Due to the sub-micron size dimensions, it is unlikely that the temperature can be measured using conventional thermometric tools, such as thermal imaging cameras or infrared detectors [34,35]. To overcome this problem, Pt is chosen for its excellent chemical stability and ideal temperature coefficient of resistance (TCR). TCR provides a simple way to calculate the temperature based on the input voltage and resistance values [15,36]. Since the device must operate at high operating temperatures, an additional layer of titanium (Ti) is required to improve Pt adhesion to the ONO layer stack [37].

### 2.3. NHP Fabrication Process

**Figure 2:**
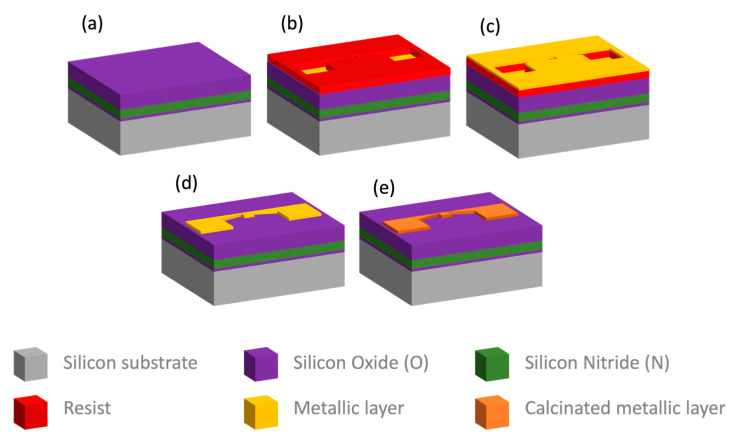
Schematic diagram of the fabrication process: (**a**) ONO stack layer preparation; (**b**) EBL exposure and development; (**c**) Metallic layer deposition (Pt and Ti); (**d**) Metal lift-off; (**e**) Electrode calcination and metallization.

In this work, the geometry of the NHP designed by KLayout software (ver. 0.27.10) is shown in Figure 1. Firstly, the positive resist PMMA (PMMA A7, Micro Chem, Newton, MA, USA) is spin coated on the clean prepared wafer substrate at a speed of 4000 rpm using a coating machine, followed by a soft bake at 180 °C for 60 s. Secondly, the substrate is transferred to a Tescan Mira-like system equipped with a pattern generator and a beam blanker for patterning. The EBL exposure is performed with the following parameters: a beam of a 500 pA current, with an energy of 30 keV and doses of up to 850 μC/cm^2^ is used to define the layout of the NHP. After this exposure step, the development process is run in an MIBK solution (Sigma Aldrich, St. Louis, MO, USA) diluted as 1:3 in isopropanol for 30 s, followed by 30 s in isopropanol as a stopper solution. This is followed by a hard bake at 100 °C for 60 s to ensure proper substrate preparation prior to evaporation. An electron beam evaporation system (Ulvac EBX-16C with Ferrotec EV S-6 e-gun, ULVAC technologies, Inc., Kanagawa, Japan) is used to deposit a 10 nm Ti adhesion layer and a 100 nm Pt layer in high vacuum. There, the lift-off process is performed in an acetone solution at 40 °C, followed by rinsing the wafer in isopropanol. Finally, the substrate is processed in the furnace (Expertech CTR 200, Expert Semiconductor Technology, Inc., Scotts Valley, CA, USA) for calcination and metallization at 650 °C for 2 h in a N_2_ environment. The entire process is shown in Figure 2.

**Figure 3:**
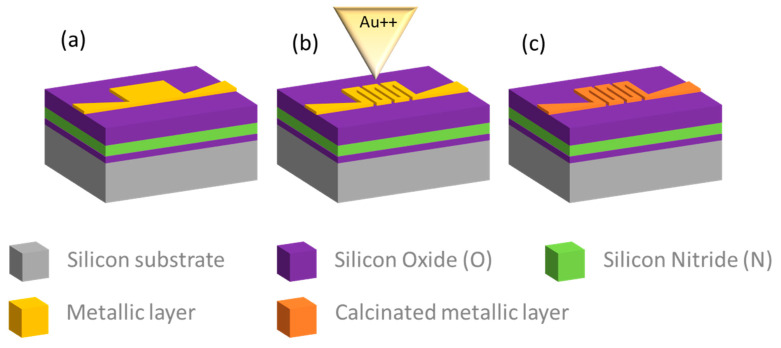
The FIB milling process: (**a**) Substrate after EBL patterning and metal layer deposition; (**b**) Fine FIB milling of the heating circuit part; (**c**) Electrode calcination and metallization.

Sequentially, a focused ion beam lithography (FIB) process is used to define the heating circuit. The FIB milling routine on the platinum layer is designed as shown in Figure 3, and it is optimized in terms of doses and energies. A Raith Velion dual beam microscope (FIB-SEM) equipped with a liquid metal alloy ion source capable of producing focused Si and Au ion beams is used for this purpose. The optimal set of parameters for precise milling of the pattern is found to be 70 keV Au ions for fluences of 90 μC/cm^2^ with a 1 nA current. 

### 2.4. NHP Characterizations

The TCR of the Pt heater defined by electron beam deposition was calculated using the approach proposed elsewhere [14,18]. Specifically, the TCR was calculated using an automatic prober (Accretech UF200R, Tokyo Seimitsu Co., Ltd., Tokyo, Japan) equipped with the ATT LOW TEMP System L200T (Advanced Temperature Test Systems GmbH, Planegg, Germany). This system provides a nominal temperature stability of ±0.1 °C, an accuracy of ±0.5 °C and a uniformity (along the chuck surface) of less than 0.5%. Resistance measurements were taken at different temperatures (20 °C, 60 °C, 100 °C and 140 °C) to extract the TCR parameter. This value was then used to calculate the relationship between temperature and heater resistance. The power consumption (P) of the device, at the different temperatures, was obtained according to Ohm’s law, where P = V^2^/R

To test the NHP application in a gas sensing system, the sensing material was sputtered with a Kenosistec 800 °C at a flux of 40 sccm Ar and 3 sccm O_2_ at a 2 W power on an NHP layout, and the deposition thickness was 180 nm. The gas sensor test condition is reported elsewhere [14,18].

## 3. Results

### 3.1. Morphology Characterization 

**Figure 4:**
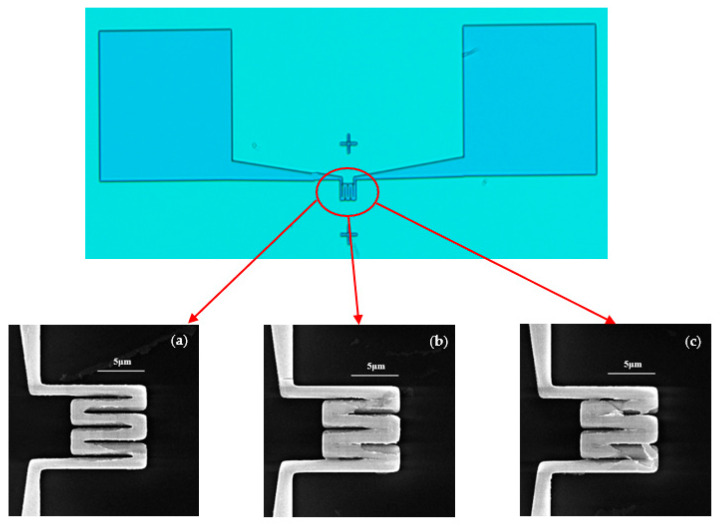
Upper, optical microscope image of a full device defined by EBL. Lower, SEM images of electrode circuits patterned by EBL at 30 keV with different electron doses of (**a**) 750 μC/cm^2^; (**b**) 800 μC/cm^2^ and (**c**) 850 μC/cm^2^.

Firstly, an attempt is made to define the full structure of the NHP using the full EBL technique; the NHP devices obtained are shown in Figure 4. In this case, the doses of 750 μC/cm^2^, 800 μC/cm^2^ and 850 μC/cm^2^ at 30 keV and 1 nA are displayed. The microscale part is clearly defined after the lift-off process of Pt and Ti for all the doses tested. However, for the NHP part, doses below 750 μC/cm^2^ are not sufficient to successfully open the area for lift-off. When the dose is increased above 750 μC/cm^2^, the gaps of the heating circuits are merged due to the proximity effect, which can cause a short circuit. 

**Figure 5:**
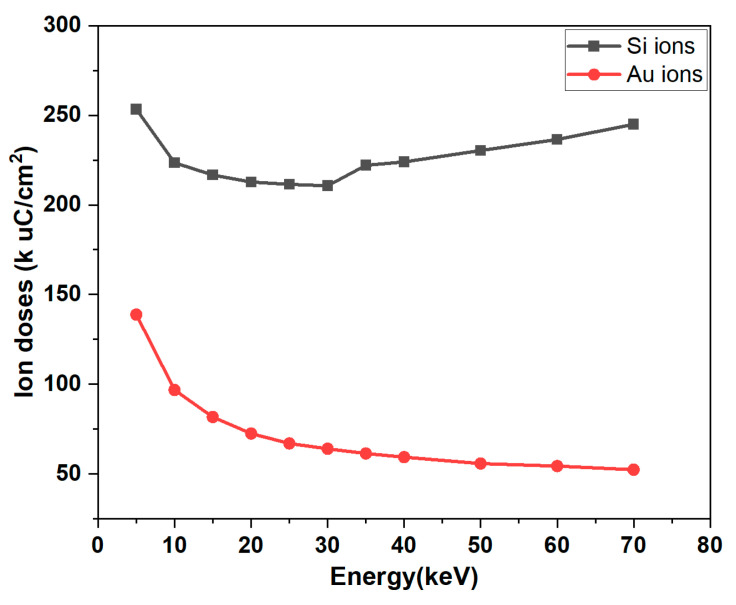
Silicon and gold ion doses versus ion energies for a 100 nm Pt film milling.

The second and successful approach explored is as follows. Instead of exposing the heating circuit only by EBL, the FIB system is used to pattern the NHP by shaping a regular rectangle of the metallic layer as shown in Figure 3. To investigate the appropriate dose and energy for FIB sputtering, an SRIM simulation is operated to reduce the window of beam parameters. The relationship between the Pt/Ti milling depth and the FIB parameters is explained as follows:

(1) $$H=\frac{\mathrm{IF}\times \mathrm{SY}\times M}{N_{A}\times \rho },$$

where H is the sputtered thickness, IF is the ion fluence (ions/cm^2^, 1 × 10^16^), and SY is the sputter yield, which is the number of atoms ejected from the target surface per incident ion and indicates the sputtering efficiency. SY takes into account the Coulomb force between the ions, and it is strictly related to the material properties, the angle of incidence and the energy of the beam [28]. M is the molar mass of the Pt atom (195.08 g/mol), ρ is the density of Pt (21.45 g/cm^3^), and N_A_ is the Avogadro constant (6.02 × 10^23^ mol^−1^). From this equation, it can be seen that the sputtered thickness depends on the ion fluence and the characteristics of the target material. Since the density of Ti (4.507 g/cm^3^) is much lower than that of Pt, the adhesion layer can be excluded from this equation. Figure 5 shows the SRIM simulation results in energy and ion doses of Si^++^ and Au^++^ for etching 100 nm of Pt.

Figure 5 shows that the ion dose required to mill a 100 nm thick platinum film varies with the energy of the beam. Compared to milling with Au ions, Si ion beams milling with the same energy require almost twice the dose to sputter the same thick Pt layer. This effect is due to the lower mass of Si ions. Since higher ion doses cause more severe lateral damage and redeposition, Au ions are the better candidate for this process. Secondly, since higher energy ion beams require lower doses and can provide better resolution due to low chromatic and space charge aberrations, a 70 keV energy is investigated. Finally, the beam current is another important factor as it defines the beam spot size. The choice of current has to be a compromise between resolution and patterning time, as high beam currents result in a large milling spot size with low resolution but fast sputtering rate [28]. In this work, the current is set to 1 nA as the lateral dimensions are not too limiting. Based on simulations, Au ions are tested with different fluences of 60, 75 and 90 k μC/cm^2^ at 70 keV. Furthermore, it is also known that sputtered material can be redeposited on the milled areas. A multiple scan approach allows for a uniform profile of the milled area by simply repeating the milling process over the same area. This approach is suitable for a box-like profile at the dame dose and reduces material residues at the bottom of the structures, thus avoiding unwanted short circuits [38]. Different scanning modes of 1 and 10 loop scanning are also investigated in this work.

**Figure 6:**
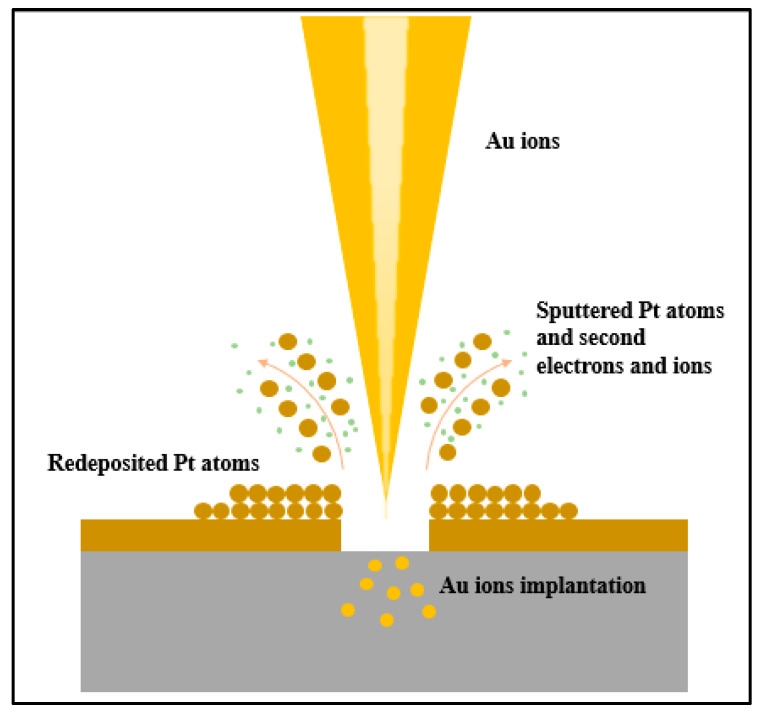
Schematic diagram of the FIB milling process.

**Figure 7:**
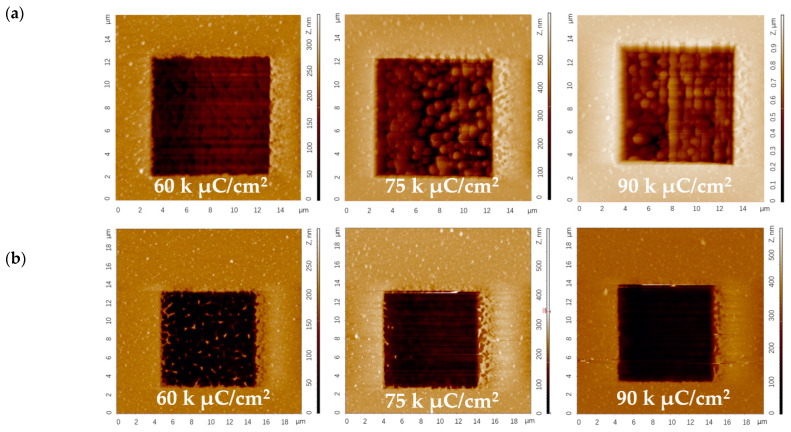
AFM images of squares milled on Pt film by Au^++^ ion beam at fluences of 60, 75 and 90 kμC/cm^2^ in (**a**) a 1-loop mode and (**b**) a 10-loop mode at a 70 keV energy.

**Figure 8:**
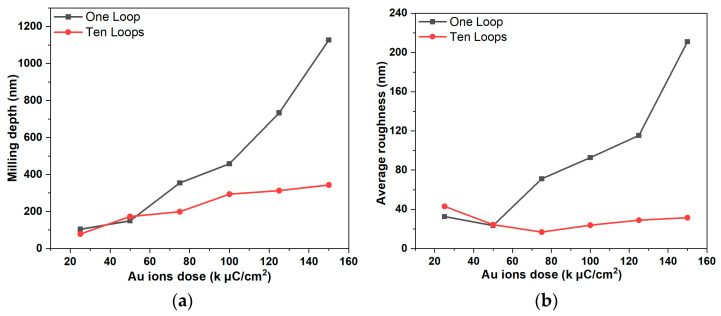
AFM results of 70 keV Au^++^ ion beam milling on Pt film with different doses and loops: (**a**) Milling depths; (**b**) Average roughness.

To verify the SRIM simulation results, an FIB is used to mill simple squares (80 × 80 μm^2^) on the surface of the Pt film by using an Au^++^ ion beam at an accelerating voltage of 35 kV (corresponding to the simulation condition of Au^+^ ions at 70 keV) with ion fluences of 60, 75 and 90 kμC/cm^2^. The milling process is shown schematically in Figure 6. The surface topography of the milled areas is characterized by AFM, and Figure 7 shows the AFM images of the FIB milling areas at different doses in 1-loop and 10-loop modes on the Pt layer. The AFM images in Figure 7a prove that the single-loop patterning process results in a deeper milling but a higher re-sputtering rate and consequently higher roughness. On the contrary, Figure 7b proves that the multi-loop milling strategy leads to a clean removal of the Pt layer, limiting the chances of redeposited Pt atoms on the milled area [28,38,39]. Figure 8 highlights the exact milling depths and roughness of the area obtained from AFM data. Figure 8 shows that the milling depth increases almost linearly with increasing ion doses for both the 1- and 10-loop milling modes. It is also clear that the single-loop approach results in deeper trenches and rougher surfaces compared to the other approach.

**Figure 9:**
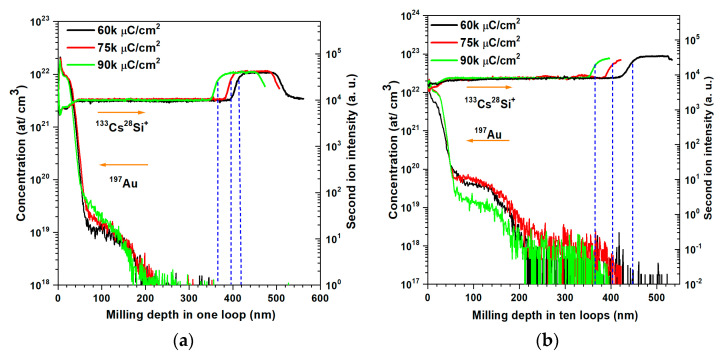
Au element distribution and Si ion intensities after Au^++^ milling by FIB on samples with different doses at (**a**) 1 loop and (**b**) 10 loops.

Secondary Ions Mass Spectrometry (SIMS) is used to examine the distribution of target surface atoms in a cross-sectional structure. Interlaced SIMS depth profiles of the deposited Pt film are shown in Figure 9. To prepare samples for SIMS analysis, a reference sample is first milled by an FIB using an Au^++^ ion beam with a concentration of 5 × 10^16^ at/cm^2^ at 35 kV. This allows correcting the sensitivity factor RSF for the measurement of the Au atomic concentration on samples. The same FIB parameters are employed with Au^++^ ion doses of 60, 75, and 90 kμC/cm^2^ at 35 kV in 1- and 10-loop milling strategies to form a 200 × 200 μm^2^ milling crater for SIMS characterization. During Au^++^ ion milling of the Pt layer, some Au^++^ is implanted into the substrate layer. The distribution of Au^++^ and the remaining Pt atoms in the cross-section area reflect the milling results.

Figure 9 shows the Au element concentration distribution and Si ion intensity in samples with different doses by 1- and 10-loop milling strategies. From the Au element concentration distribution curves, it is easy to see that the implanted Au ions are mostly located close to the crater surface with a depth of 20 nm. From the subsequent resistance measurements, it can be seen that a 20 nm thick region doped with Au atoms shows no short circuit, which proves that this FIB milling strategy can be applied to similar layout patterning. Going deeper than 20 nm, the Au concentration decreases exponentially. The CsSi^+^ second ion intensity curves reveal the milling depth by Au ions; before 350 nm of thickness, the CsSi^+^ intensity is constant except for the crater surface area. This means SIMS sputtering operating in the same layer. Then, the intensity of the CsSi^+^ second ion reaches a bump, indicating that the SIMS characterization is in another different layer. Based on the fact that the first SIMS characterization layer is greater than 350 nm and the second layer is around 100 nm, it means that by FIB milling, the sputtered depth by Au^++^ touches inside the top SiO_2_ layer. Since higher milling doses can cause a thicker bombardment area, the CsSi^+^ intensity below 90 k μC/cm^2^ shows the bump first compared to the doses of 75 and 60 kμC/cm^2^. In a one-loop milling mode, Au^++^ at 90 kμC/cm^2^ sputters 28 nm more than at 75 kμC/cm^2^, and 47 nm more than at 60 kμC/cm^2^. In a 10-loop milling mode, Au^++^ at a dose of 90 kμC/cm^2^ sputters 37 nm more than at 75 kμC/cm^2^, and 77 nm more than at 60 kμC/cm^2^. The difference in the milling depth by variable milling doses is greater in the 10-loop milling mode than in the 1-loop milling mode, which could be mainly caused by the redeposition process. At the same milling doses, the 1-loop mode can mill a thicker target layer than the 10-loop milling mode, which is in agreement with the AFM measurement results.

**Figure 10:**
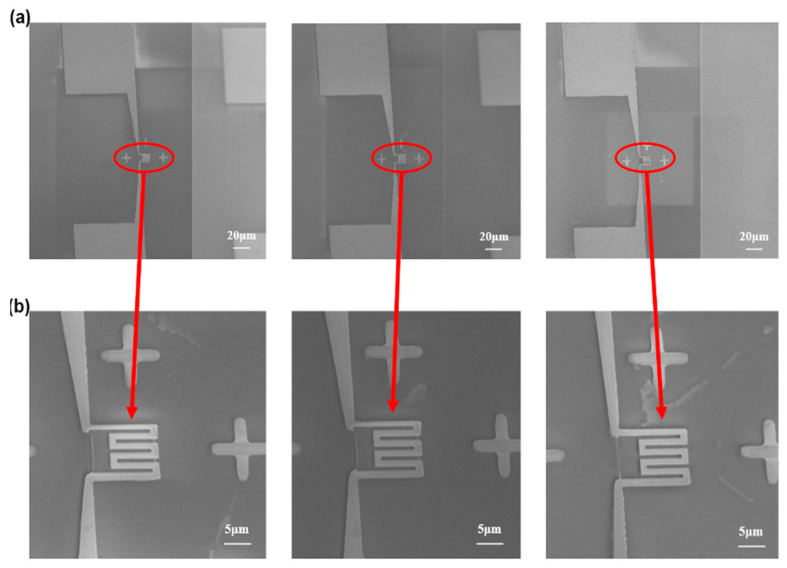
(**a**) Results of the FIB Au ion milling of the NHP by applying 60, 75 and 90 kμC/cm^2^ Au^++^ doses at 35 kV (left to right) in a 10-loop; (**b**) The corresponding electrode circuit magnified images at different doses.

Based on the AFM and SIMS characterization results, 35 kV Au ion milling with doses between 60 and 90 kμC/cm^2^ is effective for sputtering the 100 nm Pt layer for a regular structure (i.e., square). The same doses are tested for the circuit structure with nanometric gaps to evaluate the possible redeposition effects [27]. Figure 10 shows a comparison of the results for the 10-loop milling strategy by varying the dose from 60 to 90 kμC/cm^2^ by scanning electron microscopy (SEM). Smaller doses in Figure 10a,b show small connections among the heating circuit gaps causing short circuits. However, Figure 10b shows that the highest tested dose, 90 kμC/cm^2^, is required to completely remove all the connections, resulting in the desired structure.

### 3.2. Electrical Performance Measurement

**Figure 11:**
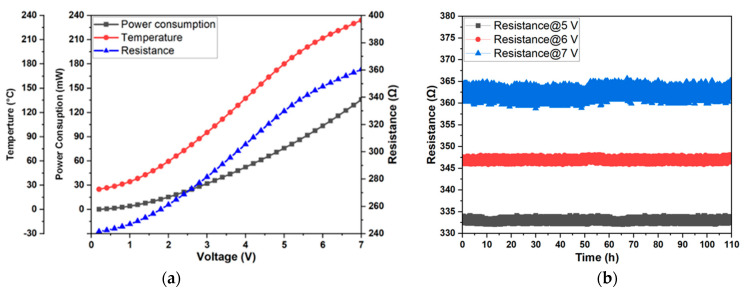
(**a**) The relationship between input voltage with working temperature and power consumption; (**b**) The stability of the NHP at different input voltages over 110 h.

Miniaturized NHP is designed for MOS gas sensor application to improve its power efficiency. Therefore, the temperature and power consumption performance measurements are performed to investigate the relationship between the input voltage and temperature/power consumption (Figure 11a). The resistance measurement of the NHP fabricated by using 35 kV Au^++^ with doses of 90 k μC/cm^2^ is carried out by Karl Suss manual probing station PM8 (SUSS MicroTec Semiconductor, Garching, Germany), equipped with an Agilent 4156C Precision Semiconductor Parameter Analyzer (Agilent Technologies, Santa Clara, CA, USA). The temperature of the NHP caused by Joule heating is determined by using the TCR measurement term of Pt, which is as follows:

R_T_ = R_0_ (1 + 0.0025 T), (2)

where R_T_ is the resistance of NHP at a given voltage, R_0_ is the resistance of the NHP at 0 °C, and T is the current temperature [17,18]. 

The TCR determines the rate at which the Pt resistance varies as a function of the environmental temperature, and it is calculated as described in Section 2.4 [14,18], resulting in 0.0025 °C^−1^. When 0.05 V is applied to the NHP, the resistance is 241.64 Ω, which is considered to be the resistance at room temperature. To calculate R_0_, the following equation is used: 241.64/(1 + 0.0025 × 25 °C), which is 227.43 Ω. Using this equation, the temperature is calculated and shown in Figure 11 from 0.1 V to 7 V. The resistance of the NHP changing trend is consistent with the temperature changing trend due to their linear relationship revealed by the equation by increasing the input voltage. The rate of increase in temperature and resistance slows down, but the rate of increase in power is faster when the input voltage is higher, which is caused by the accelerated thermal diffusion at high temperature. The power consumption (P) of the device at the different temperatures was obtained using Ohm’s law, where P = V^2^/R. Table 1 shows a comparison between the power consumption of the NHP with that of the state-of-the-art devices produced by micro- and nanofabrication approaches.

As can be seen in Table 1, the power consumption of the NHP is higher than that of micro hotplates fabricated over suspended membranes or bridges, but much lower than that of micro-hotplates produced over bulk substrates due to the very small dimensions of our device. Therefore, considering the current state of the art in the nano- and micro-hotplate field, we demonstrated that by using advanced nanofabrication technologies such as FIB and EBL, it is possible to develop scaled hotplates that show low power consumption while preserving the great advantage of the robustness of the bulk substrate, without the need to define a suspended membrane or bridge, which have low mechanical strength.

For resistance repeatability, the test environment is maintained at a constant relative humidity of 40% RH and a room temperature of 25 °C with different input voltages (5, 6 and 7 V) for 110 h. The resistance values of NHP at different input voltages are shown in Figure 11b. When the operating voltage at the NHP is 5 V, the resistance change range is 2 Ω, indicating a resistance drift of 0.6% and a temperature error of 3.5 °C. When the voltages are 6 V and 7 V, the resistance drifts are 0.7% and 1.4% respectively, and the temperature errors are 4.39 °C and 8.79 °C, respectively.

### 3.3. NHP Application for ZnO Gas Sensor Device

**Figure 12:**
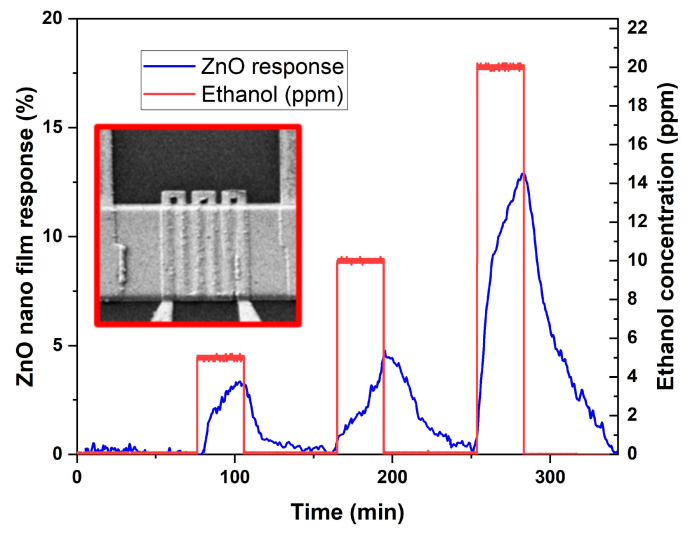
The response of the ZnO sensing film deposited on the NHP (as shown in the inset) at 5 V and 250 °C towards the target gas of ethanol at concentrations of 5 ppm, 10 ppm and 20 ppm.

To test the performance of the sensor based on the NHP developed above, the structure of the gas sensor is completed by depositing two Ti/Pt electrodes on the device by e-beam evaporation. A sensing layer of ZnO is then deposited on the surface of the device by using magnetron sputtering, and the deposition pattern is defined by a lift-off process. Specifically, the PMMA resist is spin-coated onto the silicon wafer. EBL exposure is then performed at an accelerated voltage of 20 kV to define the ZnO deposition area. After resist development, the ZnO film is sputtered onto the device surface magnetron deposition. After lift-off, the wafer is treated in a pure N_2_ environment at 650 °C for 2 h for calcinating the ZnO film. Finally, the resulting device (inset of Figure 12) is tested toward the target gas for sensing application. The sensing performance is tested in the gas test bench against different concentrations of ethanol, keeping the temperature (22 °C) and the relative humidity RH% = 40%) in the gas chamber constant. Synthetic air (20%O_2_ and 80%N_2_) and target gas are injected from certified cylinders (N5.0 degree of purity) through mass-flow controllers with a total flow of 200 sccm. The results of the characterization are shown in Figure 12. The baseline resistance (R_air_) of the sensing material in air is defined as the background reference, and the obtained resistance of the sensing material in the target gas is defined as R_gas_. Therefore, the change in resistance of the sensing material under the target gas is proportional to the gas sensing response, which is calculated using the following equation:

(3) $$\mathrm{Response}\;(\%)=\left((\frac{R_{\mathrm{air}}}{R_{\mathrm{gas}}}{)}^{-1}-1\right)\times 100.$$

The target gas injection time is 30 min, and the stabilization time is 60 min. The ZnO nanofilm shows positive correlation responses toward ethanol with increasing concentrations. In addition, response/recovery times are calculated for the detection of 20 ppm of ethanol. In particular, response time is considered to be the time required to reach 90% of the response value, while the recovery time is considered to be the time required to recover 90% of the baseline signal. On this basis, the response and recovery times are approximately 20 and 35 minutes, respectively.

## 4. Conclusions

In this work, a nanoscale hotplate was fabricated by combining EBL and FIB technologies, where EBL was used to define the micro-size pad parts, and an FIB was used to pattern the submicron elements of the heating circuit on the Pt heater area. Based on SRIM software simulation, different Au^++^ doses were tested under different milling modes, and the FIB sputtering results were characterized by SEM, AFM and SIMS. The results revealed that the most suitable Au^++^ dose was 90 kμC/cm^2^ at a 70 keV energy. In addition, the 10-loop milling strategy produced a higher quality sputtered surface than the 1-loop milling mode. The resistance of the NHP was measured at different voltages, and the corresponding temperature and power consumption were calculated based on Pt TCR value. The resistance repeatability of the NHP was investigated at different high voltages of 5, 6 and 7 V for 110 h. During this time, the resistance shifts were 0.6%, 0.7% and 1.4% at voltages of 5, 6 and 7 V, respectively. Finally, the application of the NHP to the ZnO-based gas sensor was investigated, which showed an increasing response to ethanol with increasing concentrations at 5 V (250 °C) and 40 RH% humidity.

## Figures and Tables

**Table 1:** Comparison between the specifications of the NHP developed in this work and state-of-the-art examples.

Hotplate Dimensions (μm^2^)	Hμhotplate Geometry	Temperature (°C)	Power (mW)	Reference
100 × 100	Suspended membrane	300	5.85	[13]
1327 × 1327	Suspended membrane	450	107	[14]
250 × 250	Suspended bridge	100	35	[15]
280 × 290	Suspended membrane	100	60	[16]
1300 × 1300	Suspended membrane	250	50	[17]
300 × 300	Bulk alumina	250	770	[40]
11 × 11	Bulk silicon	230	130	This work

## Data Availability

The data presented in this study are available on request from the corresponding authors.
